# Novel pyrrolo-1,5-benzoxazepine compounds display significant activity against resistant chronic myeloid leukaemia cells *in vitro*, in *ex vivo* patient samples and *in vivo*

**DOI:** 10.1038/sj.bjc.6605670

**Published:** 2010-04-20

**Authors:** S A Bright, A M McElligott, J W O'Connell, L O'Connor, P Carroll, G Campiani, M W Deininger, E Conneally, M Lawler, D C Williams, D M Zisterer

**Affiliations:** 1 School of Biochemistry and Immunology, Trinity College, Dublin 2, Ireland; 2 Institute of Molecular Medicine, St. James's Hospital, Trinity College, Dublin, Ireland; 3 Cancer Clinical Trials Office, St. James's Hospital, Dublin, Ireland; 4 European Research Centre for Drug Discovery and Development, Universita’degli Studi di Siena, Siena, Italy; 5 Division of Hematology and Medical Oncology, Oregon Health and Science University, Portland, OR, USA

**Keywords:** PBOX, CML, T315I, Bcr-Abl, apoptosis

## Abstract

**Background:**

Imatinib is a direct and potent inhibitor of the constitutively active tyrosine kinase, breakpoint cluster region-Abelson (Bcr-Abl), which is central to the pathogenesis of chronic myeloid leukaemia (CML) patients. As such, imatinib has become the front-line treatment for CML patients. However, the recent emergence of imatinib resistance, commonly associated with point mutations within the kinase domain, has led to the search for alternative drug treatments and combination therapies for CML.

**Methods:**

In this report, we analyse the effects of representative members of the novel pro-apoptotic microtubule depolymerising pyrrolo-1,5-benzoxazepines or PBOX compounds on chemotherapy-refractory CML cells using a series of Bcr-Abl mutant cell lines, clinical *ex vivo* patient samples and an *in vivo* mouse model.

**Results:**

The PBOX compounds potently reduce cell viability in cells expressing the E225K and H396P mutants as well as the highly resistant T315I mutant. The PBOX compounds also induce apoptosis in primary CML samples including those resistant to imatinib. We also show for the first time, the *in vivo* efficacy of the pro-apoptotic PBOX compound, PBOX-6, in a CML mouse model of the T315I Bcr-Abl mutant.

**Conclusion:**

Results from this study highlight the potential of these novel series of PBOX compounds as an effective therapy against CML.

Chronic myeloid leukaemia (CML) is caused by breakpoint cluster region-Abelson (Bcr-Abl), a constitutively active tyrosine kinase that is the result of the reciprocal translocation between chromosome 9 and 22, t(9;22), cytogenetically visible as the Philadelphia chromosome (Deininger *et al*, 2000). The disease is thought to originate from the transformation of a haematological stem cell. Bcr-Abl-positive cells are resistant to apoptosis due to the continuous activation of signalling pathways downstream of Bcr-Abl, including the signal transducer and activator transcription and phosphatidyl inositol-3 kinase pathways. The majority of CML patients are diagnosed in the chronic phase that is easily managed with the novel kinase inhibitor, imatinib (imatinib mesylate; STI571; Gleevec), which directly targets the oncogenic Bcr-Abl protein (Deininger *et al*, 2005). However, some patients acquire resistance to imatinib and progress to a more aggressive phase of disease termed the accelerated phase and eventually the blastic phase, which resembles an acute leukaemia that is largely resistant to imatinib and rapidly fatal (Deininger and Druker, 2003). Even in patients reaching a complete cytogenetic response, residual disease remains detectable by PCR techniques suggesting a continued threat of relapse (O’Hare *et al*, 2008).

There are many different mechanisms through which patients become resistant to imatinib, including an increase in the levels of Bcr-Abl protein expression levels through amplification of the Bcr-Abl gene, an efflux of imatinib through multi-drug resistant (MDR) proteins such as P-glycoprotein (P-gp) (Mahon *et al*, 2000) and activation of alternative signalling pathways downstream of or independent to Bcr-Abl (Capdeville *et al*, 2002). Progression of the disease can also be associated with additional mutations in oncogenes such as p53, Ras and retinoblastoma gene. However, the major mechanism of imatinib resistance is due to mutations at critical points in the Bcr-Abl gene. These Bcr-Abl mutations can occur in several functionally distinct regions within the kinase domain and confer varying degrees of insensitivity to imatinib. More than 60 different amino-acid substitutions have been found to date and many of these are mutated at more than one alternative residue (Apperley, 2007a, 2007b). Mutations fall into two groups, those that alter amino acids that directly contact imatinib and those that prevent Bcr-Abl from achieving the inactive conformational state required for imatinib binding. Of the mutations that result from amino-acid substitutions, the T315I and E255K mutations are among the most prevalent (Hughes *et al*, 2006). Crucially the T315I mutation is also associated with the highest degree of imatinib resistance, it prevents the formation of a critical hydrogen bond between imatinib and Bcr-Abl and changes the conformation of Bcr-Abl to prevent imatinib binding.

These mutations raise the possibility that additional drugs that target CML cells through alternative mechanisms to Bcr-Abl have the potential to be successful in treating imatinib-resistant CML patients (Druker *et al*, 2001). As a result, a number of studies have been conducted with imatinib in combination with other anticancer therapeutics including farnesyltransferase inhibitors such as SCH66336 (lonafarnib) (Peters *et al*, 2001; Nakajima *et al*, 2003; Borthakur *et al*, 2006), mitogen-activated protein kinase pathway inhibitors PD184352, PD98059 and U0126 (Yu *et al*, 2002; Nguyen *et al*, 2006) and histone deacetylase inhibitors MS-275 and SAHA (suberoylanilide hydroxamic acid) among others (Kelly *et al*, 2003; Yu *et al*, 2003; Fiskus *et al*, 2006a) with varying degrees of success in *in vitro* and *in vivo* animal models. Nilotinib in combination with LBH589 also produced synergistic results (Fiskus *et al*, 2006b).

Another approach to overcome imatinib resistance has led to the development of novel second-generation Bcr-Abl tyrosine kinase inhibitors. Of these, dasatinib, an orally active, small molecule that inhibits Bcr-Abl, along with multiple other kinases including the SRC family kinases (Guilhot *et al*, 2007) and nilotinib (Tasigna; Novartis Pharmaceuticals, Basel, Switzerland), a phenyl amino-pyrimidine analogue of imatinib, have recently been approved by the FDA for treatment of imatinib-resistant CML patients (McFarland and Wetzstein, 2009). Although these second-generation tyrosine kinase inhibitors are effective against many Bcr-Abl mutants, they are completely ineffective against the T315I mutants. Although third-line tyrosine kinase inhibitors with activity against T315I mutants are in clinical trials, it is likely that Bcr-Abl-independent resistance will arise in advanced CML, suggesting that agents that target CML cells through alternative mechanisms to Bcr-Abl have the potential to be successful in treating imatinib-resistant CML patients (Quintás-Cardama and Cortes, 2008b).

Recently, some members of a novel set of compounds, the pyrrolo-1,5-benzoxazepine (PBOX) compounds, have been shown to induce apoptosis in a wide range of solid tumours and haematological malignancies (Zisterer *et al*, 2000). Tubulin has been identified as the molecular target of these compounds that induce apoptosis in CML cells by bypassing Bcr-Abl (Mc Gee *et al*, 2001). The PBOX compounds have also shown efficacy in *ex vivo* chronic lymphocytic leukaemia (CLL) samples (McElligott *et al*, 2009) and in an *in vivo* mouse mammary carcinoma model (Greene *et al*, 2005). Furthermore, we have also shown that PBOX-6 in combination with imatinib enhances apoptosis in imatinib-resistant CML cell lines that overexpress the Bcr-Abl protein (Greene *et al*, 2007). It was therefore of interest to determine if the PBOX compounds could similarly induce/enhance apoptosis in CML cells that contain the highly resistant T315I mutation both *in vitro* and *in vivo*.

Using a series of Bcr-Abl mutant cell lines (O’Hare *et al*, 2008), we showed the ability of PBOX-6 and PBOX-15 to reduce viability in cells that contain the imatinib-resistant mutations E225K, H396P and T315I *in vitro*. The PBOX compounds also induced apoptosis in clinical *ex vivo* CML patient samples including those resistant to imatinib whereas PBOX-6 reduced tumour load in a CML mouse model containing the T315I Bcr-Abl mutation. We conclude that the pro-apoptotic PBOX compounds have potential as novel therapeutics in the treatment of CML patients, including those harbouring the notorious T315I mutation.

## Design and methods

### Cell culture

The murine pro-B cell lines, Baf/3 cells, transfected with either the native or mutated versions of Bcr-Abl, were previously described (O’Hare *et al*, 2008). Baf/3 cell lines were grown in RPMI-1640 (GlutaMAX) medium supplemented with 10% (v/v) fetal bovine serum (FBS) and 50 *μ*g ml^−1^ penicillin/streptomycin. Parental Baf/3 cells also required 10 ng ml^−1^ IL-3. Cells were grown in a humidified environment maintained at 95% O_2_ and 5% CO_2_ and passaged at least twice weekly. Cells were seeded at a density of 2 × 10^5^ cells per ml.

### Reagents

RPMI-1640 medium was obtained from Biosciences (Dublin, Ireland) and FBS from Invitrogen (Paisley, UK). The enhanced chemiluminescence reagents were supplied by Amersham Biosciences (Buckinghamshire, UK). The BCA reagents were purchased from Pierce (Rockford, IL, USA) and polyvinylidene difluoride membranes were sourced from Millipore (Cork, Ireland). Actin and Bcr-Abl antibodies were obtained from Calbiochem (Nottingham, UK) whereas the CD45 antibody was from BD Biosciences (Oxford, UK). Annexin V was obtained from IQ Products (Groningen, the Netherlands), Lymphoprep from Axis-Shield (Kimbolton, Cambridgeshire, UK) whereas the binding buffer and AlamarBlue dye were purchased from Biosource (London, UK). Imatinib was provided by Novartis Pharmaceuticals. All other reagents were purchased from Sigma (Tallaght, Dublin, Ireland).

### AlamarBlue viability assay

Baf/3 cells expressing native or mutant Bcr-Abl were seeded in 200 *μ*l medium in a 96-well plate. Cells were treated and incubated as required. AlamarBlue (20 *μ*l) was added to each well and incubated at 37°C in the dark for 3 h. Plates were then read on a fluorescent plate reader (SpectraMax Gemini; Molecular Devices, Wokingham, Berkshire, UK) with excitation and emission wavelengths of 544 and 590 nm, respectively. Experiments were performed in triplicate. A sample containing only reagent and medium was used as a blank. Vehicle samples were set as 100% viability from which any decrease in viability was calculated.

### Flow cytometry

Baf/3 cells expressing native or mutant Bcr-Abl were seeded in 12-well plates and treated with the designated compound/s or corresponding vehicle. After the required treatment time samples were centrifuged at 500 **g** for 5 min. Cells were then resuspended in 100 *μ*l ice-cold phosphate-buffered saline (PBS) and ice-cold 70% (v/v) ethanol (1 ml) and stored overnight at 4°C. Cells were subsequently pelleted at 800 **g** for 10 min and resuspended in 200 *μ*l PBS. RNase A (10 *μ*g ml^−1^) and propidium iodide (100 *μ*g ml^−1^) were then added to each sample and incubated for 30 min at 37°C in the dark. Cell-cycle analysis was performed at 488 nM using a Becton Dickinson (Franklin Lakes, NJ, USA) FACS Calibur flow cytometer. Apoptosis was determined by quantification of the pre-G1 peak. CellQuest was then used to analyse the data of 10 000 gated cells once cell debris had been excluded. The data was stored as frequency histograms (Riccardi and Nicoletti, 2006).

### Western blot analysis

SDS–polyacrylamide gel electrophoresis was performed as previously described (Bright *et al*, 2009). Briefly, whole-cell lysates were prepared in Laemmli buffer and run on an 8% gel. Equal protein loading was ensured by performing a BCA protein determination assay. Samples were then transferred to polyvinylidene difluoride membranes overnight, blocked in 5% (w/v) marvel in Tris-buffered saline (pH 7.6)/0.05% Tween 20, incubated for 1 h with an anti-Abl or anti-Actin mAb, washed, incubated for 1 h with a horseradish peroxidase-linked secondary Ab and washed again. Blots were developed using enhanced chemiluminescence and an automated developer (Fuji X-ray film processor, Fuji-Glasnevin, Dublin, Ireland).

### *Ex vivo* CML patient samples and annexin V staining

Peripheral blood (10 ml) was collected with informed consent from newly diagnosed treatment naive (*n*=4) or blast crisis imatinib-resistant CML patients (*n*=2) in EDTA-anticoagulant tubes. Ethical approval for all work carried out on *ex vivo* CML patient samples was obtained from the St. James's Hospital and Adelaide and Meath incorporating the National Children's Hospital Ethics Committee. Fresh blood was diluted 1 : 3 with unsupplemented RPMI medium and carefully added to half the equivalent volume of Lymphoprep. Samples were centrifuged at 750 **g** for 30 min to form a Ficoll gradient. The white buffy layer containing the white blood cells (WBCs) (or peripheral blood mononuclear cells) was carefully removed, diluted to a volume of 50 ml with medium and centrifuged again for 10 min at 650 **g**. White blood cells were then seeded at a density of 1 × 10^6^ cells per 1 ml medium in RPMI medium supplemented with 10% (v/v) FBS, 1% (v/v) L-glutamine and 50 *μ*g ml^−1^ penicillin/streptomycin. After the required incubation, cells were centrifuged at 650 **g** for 5 min, incubated in the dark at room temperature for 10 min with anti-CD45 antibody, centrifuged as before, washed in 1 × annexin V binding buffer (1 ml), centrifuged, labelled with Annexin V antibody on ice for 15 min in the dark, washed and finally resuspended them in 500 *μ*l Annexin V binding buffer. Leukaemic cells were then identified and gated by flow cytometry based on their low to medium side scatter and low CD45 expression, and apoptosis was analysed by annexin V staining (Lacombe *et al*, 1997).

### Mice

Specific pathogen-free 6- to 8-week-old adult female BALB/c-nu/nu mice were purchased from Harlan Laboratories (Cambridgeshire, UK). Mice were housed in sterile individually ventilated cages, food and water were provided *ad libitum*, in accordance with the standard operating procedures set down in SI 17/94 of the European Union. Mice were acclimatised for 1 week before the initiation of any *in vivo* experiments. Animals were killed by CO_2_ if found to be in distress (hunching, failure to groom etc.) or if tumour volume exceeded 10% of mouse bodyweight. Ethical approval was obtained by the BioResources Committee, Trinity College, Dublin.

### *In vivo* tumour studies

The *in vivo* antitumour efficacy of PBOX-6 was examined using a Baf/3 xenograft containing the T315I mutant of Bcr-Abl in a BALB/c mouse model. Female BALB/c-nu/nu mice were inoculated subcutaneously (s.c.) into the right flank with 3 × 10^6^ Baf/3 cells in the log phase of growth. After 8 days, we observed that tumours had reached approximately 4 mm in diameter and we randomly divided mice into two groups. A stock solution of PBOX-6 was prepared at a concentration of 15 mg ml^−1^ in 1 : 1 ethanol/cremophor EL. When required, this was then diluted 1 in 5 in sterile PBS to give a final concentration of 7.5 mg kg^−1^ or 0.15 mg per mouse. The treatment group (*n*=6) received an intratumoural (i.t.) injection (Williams *et al*, 2007; Shah *et al*, 2009) of PBOX-6 once daily for the duration of the experiment starting on day 8. Control mice (*n*=6) received a single daily i.t. injection of 50 *μ*l of the equivalent vehicle also starting on day 8. Tumour growth was measured every second day with a sterile vernier calliper. The long (*L*) and short (*S*) axes were recorded, and tumour volume (*V*) was calculated using the following equation as described previously (Beck *et al*, 2003). 
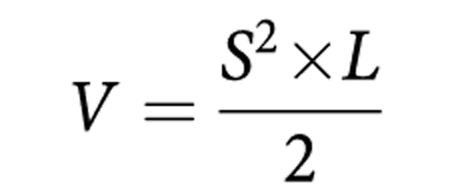


Mice were killed by CO_2_ asphyxiation at the experimental end point or if found to be under any excess duress.

### Statistical analysis

The software Prism GraphPad (La Jolla, CA, USA) was used to carry out statistical analysis when comparing two samples using the Student's paired (*ex vivo* studies) or unpaired (*in vivo* studies) *t*-test. For illustrative purposes, the *P*-values are presented as *P*<0.05, ^*^ and *P*<0.01, ^**^ respectively.

## Results

### Mutations in the Bcr-Abl gene confer varying degrees of resistance to imatinib in transfected Baf/3 cell lines

**Figure 1:**
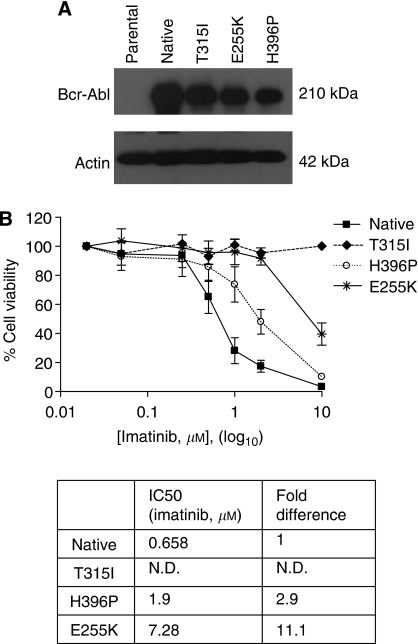
Mutant Baf/3 cell lines containing the Bcr-Abl protein display varying degrees of resistance to imatinib when compared with the native cells. (**A**) Baf/3 cells were seeded during the log phase of growth and incubated for 24 h. Samples were prepared for SDS–polyacrylamide gel electrophoresis and run on an 8% gel. Polyvinylidene difluoride membranes were incubated overnight with an anti-Bcr-Abl mAb. Actin is shown as a loading control. Results are representative of three independent experiments. (**B**) Cells were treated for 48 h with a vehicle (Veh) (0.01 % DMSO) or a range of imatinib concentrations (50 nM–10 *μ*M) in a 96-well plate. After the incubation period, we added AlamarBlue dye (20 *μ*l) to each well and incubated samples for 3 h. Cells were subsequently analysed for cell viability. Values represent the mean±s.e.m. of three independent experiments performed in triplicate. ND, not determined.

The status of the Bcr-Abl protein in parental and transfected Baf/3 cells was confirmed by subjecting untreated cells to SDS–polyacrylamide gel electrophoresis and western blotting. Results confirmed the presence of the Bcr-Abl protein in native cells and each of the mutant cell lines and absence of the protein in the parental, non-transformed Baf/3 cells (Figure 1A).

An AlamarBlue assay was used to show the varying effects of imatinib on each cell line. As expected, imatinib displayed the highest level of toxicity against the native cell line followed by the H396P, E255K and finally the T315I mutant cell lines. Specifically, native cells were found to have an IC_50_ value of 658 nM towards imatinib. H396P cells were 2.9 times more resistant to imatinib than native cells whereas E255K cells were 11.1 times more resistant. The IC_50_ value for T315I cells could not be determined as these cells were found to be completely resistant to imatinib even at concentrations as high as 10 *μ*M (Figure 1B).

### PBOX compounds significantly reduce cell viability and induce apoptosis in imatinib-resistant and native Baf/3 cell lines

**Figure 2:**
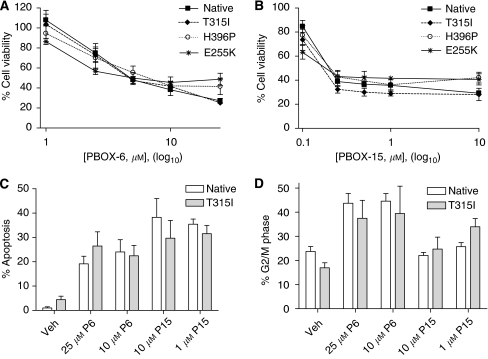
PBOX-6 and PBOX-15 reduce cell viability and induce apoptosis equipotently in native and T315I Baf/3 cell lines. Native and Bcr-Abl mutant Baf/3 cells were seeded during the log phase of growth and treated for 48 h with a vehicle (1% (v/v) ethanol) (Veh) or with the indicated concentrations of PBOX-6 or PBOX-15 (P6, P15). After the incubation period, we assessed cells for (**A** and **B**) cell viability by the AlamarBlue viability assay, (**C**) apoptosis by quantification of the pre-G1 peak and (**D**) the percentage of cells in the G2/M phase. Values represent the mean±s.e.m. of three independent experiments performed in triplicate.

The pro-apoptotic PBOX compounds were found to significantly reduce cell viability in all cell lines examined. Viability was reduced to 27.2±2.4, 25.2±0.8, 41.5±8.1 and 48.7±6.1% in native, T315I, H396P and E255K cells treated with 25 *μ*M PBOX-6 (Figure 2A). PBOX-15 also significantly reduced cell viability in all the Bcr-Abl mutant Baf/3 cells. Specifically, PBOX-15 (10 *μ*M) reduced viability to 29.2±1.9, 28.1±5.1, 42.1±4.5 and 40.7±4.6% in native, T315I, H396P and E255K, respectively (Figure 2B).

As the PBOX compounds caused similar effects against all the mutations tested, it was decided to focus our attention on the T315I mutation that conferred the highest degree of resistance to imatinib. Flow cytometry results showed that PBOX-6 and PBOX-15 induced similar levels of apoptosis (Figure 2C) in both native and T315I cells. PBOX-6 also caused a similar number of cells to undergo a sustained G2/M arrest, with the percentage of cells in the G2/M phase increasing from 23.7±2.1 and 16.9±2.1% to 43.7±4.0 and 37.5±7.5% in WT and T315I cells, respectively (Figure 2D).

The PBOX compounds were also evaluated for their effects on parental non-transfected Baf/3 cells, whereas the compounds did induce some toxicity on these cells (data not shown) it should be noted that the cells had been artificially stimulated using IL-3 to produce actively dividing cells. However, B cells *in vivo* are not actively cycling and are therefore unlikely to be affected by the compounds.

### PBOX compounds induce apoptosis in *ex vivo* samples from newly diagnosed and imatinib-resistant blast crisis phase CML patients

The advent of cell lines has allowed us to gain insight into many molecular signalling pathways; they are a useful tool to help develop novel therapeutic anticancer agents. However as cell lines have been engineered to continuously and consistently divide, they do not fully represent primary leukaemia cells. For this reason, it is necessary to confirm the effects of potentially novel therapeutics found in cell lines on *ex vivo* patient samples. For this, we obtained informed consent from a cohort of newly diagnosed and treatment naive CML patients along with those that had refractory disease. Annexin V staining was then used to identify apoptotic cells. Patient samples 1–4 were newly diagnosed CML patients who subsequently responded well to either imatinib or nilotinib, none have since progressed to later stages of CML. Patient samples 5 and 6 were obtained from imatinib- and dasatinib-insensitive patients in blast crisis (Table 1). Patient 5 tested 100% positive for the T315I mutation at time of death that occurred approximately 1 month after sample collection, whereas patient 6 who is currently on nilotinib treatment tested 100% positive for the F317l mutation at the time of sample collection and had a minor T315I mutant clone.

**Figure 3:**
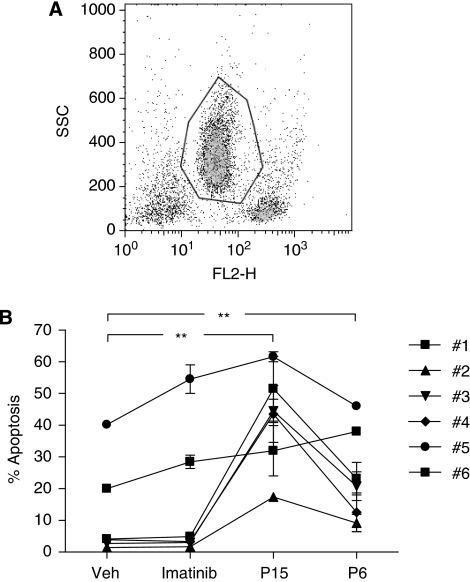
Isolation of leukaemic cells from CML patient blood samples. White blood cells were isolated from the fresh peripheral blood of CML patients (*n*=6) by Ficoll gradient. Cells were seeded at a density of 1 × 10^6^ cells per ml in 24-well plates and treated with a vehicle (Veh) (1% (v/v) ethanol or 0.0025% (v/v) DMSO), 10 *μ*M PBOX-15 (P15), 25 *μ*M PBOX-6 (P6) or 250 nM imatinib for 72 h. Cells were then labelled with an anti-CD45 antibody and FITC-annexin V and analysed by flow cytometry. Leukaemic cells were identified and gated based on their low to medium side scatter and low CD45 expression. (**A**) A representative dot plot of CD45-stained vehicle-treated cells is shown. (**B**) Percentage of cells undergoing apoptosis as determined by annexin-V-positive staining. Experiments were performed in duplicate. Statistical analysis was performed using the Student's paired *t*-test, ^**^ *P*<0.01.

White blood cells were isolated from freshly obtained blood samples, seeded and treated for 72 h with a PBOX compound (25 *μ*M PBOX-6 or 10 *μ*M PBOX-15) or 250 nM imatinib, a physiologically relevant concentration of STI571. Leukaemic cells were identified by their low to medium side scatter and low CD45 expression after annexin V staining (Figure 3A).

Collectively, when all samples were combined, PBOX-6 induced 24.7±5.0% apoptosis (*P*-value: 0.0050,^**^) whereas PBOX-15 induced 39.5±5.8% apoptosis (*P*-value: 0.0059,^**^) compared with background levels of apoptosis of 13.4±5.5% in control samples. There was no significant difference between the control and the imatinib-treated samples that underwent 18.2±7.7% apoptosis (Figure 3B). This may have been due to the inclusion of the two imatinib-resistant patient samples. No individual sample showed a lack of response to either PBOX-6 or PBOX-15.

### PBOX-6 administration significantly inhibits tumour growth in a CML mouse model of the imatinib-resistant T315I mutants

Five- to six-week-old female BALB/c-nude mice were inoculated s.c. into the right flank with 3 × 10^6^ Baf/3-T315I cells in the log phase of growth. Tumours started to appear by day 5/6 and were measurable by day 8. Mice were divided into groups with small-, medium- and larger-sized tumours and then further randomly divided into two groups (*n*=6) to ensure the average tumour size was equal for both groups. One group received daily i.t. injections of 7.5 mg kg^−1^ PBOX-6 whereas the other group received a vehicle (10% (v/v) ethanol and 10% (v/v) cremophor EL in PBS).

**Figure 4:**
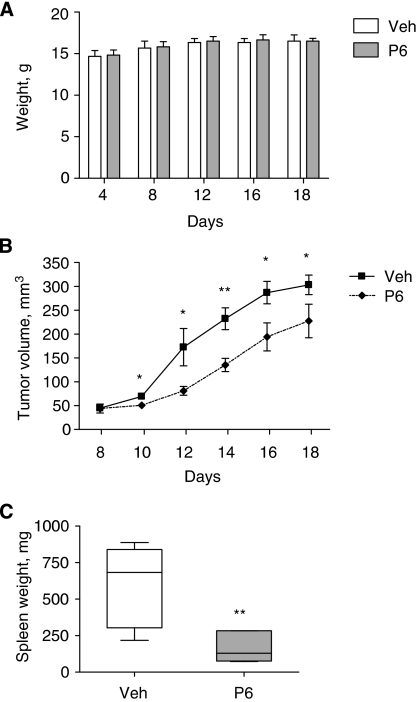
Administration of PBOX-6 significantly inhibits tumour growth and prevents gross spleen enlargement in an *in vivo* mouse model of CML harboring the T315I mutation. Five- to six-week-old female BALB/c-nude (nu/nu) mice were inoculated s.c. into the right flank with 3 × 10^6^ Baf/3-Bcr-Abl-T315I cells in the log phase of growth. When tumours had reached a diameter of ∼4 mm (day 8), mice were randomly divided into two treatment groups (*n*=6) and received daily i.t. injections of PBOX-6 (P6) (7.5 mg kg^−1^) or a vehicle (Veh) (10% (v/v) cremophor, 10% (v/v) ethanol in PBS). (**A**) Mice were weighed every 4 days and at the experimental end point (day 18). (**B**) Tumour growth was measured every second day, the long (*L*) and short (*S*) axes were recorded, and tumour volume (*V*) calculated using the equation: *V*=(*S*^2^ × *L*)/2 (Beck *et al.*, 2003). (**C**) Mice were killed on day 18, spleens were removed and weighed. Statistical analysis was performed using the Student's unpaired *t*-test, ^*^P<0.05; ^**^P<0.01.

Results showed that PBOX-6 did not adversely affect weight. In fact, both groups put on a small amount of weight during the experiment increasing from 14.7±0.7 and 14.8±0.6 g to 16.5±0.8 and 16.5±0.3 g in control and PBOX-treated groups, respectively. This would be expected with healthy mice of such an age (Figure 4A). Close inspection of mice also ensured that no mouse suffered any form of distress such as failing to groom, hunching.

Tumour growth was measured every second day, the long (*L*) and short (*S*) axes were recorded, and tumour volume (*V*) calculated using the following equation: *V*=(*S*^2^ × *L*)/2 (Beck *et al*, 2003). Injections were initiated on day 8 when there was no significant difference in tumour volume between the control and the PBOX-treated groups. By day 10, PBOX-6 had significantly reduced tumour growth when compared to control group. Tumour burden was continuously and significantly inhibited up until the experimental end point (Figure 4B). Statistical analysis was performed using an unpaired Student's *t*-test.

At the experimental end point, day 18, mice were killed by CO_2_ asphyxiation and a splenectomy was performed. The vehicle-treated group showed gross enlargement of spleens, or splenomegaly a clinical symptom of advanced disease, when compared to the PBOX-6-treated group, with a 3.7-fold increase in weight from 163.3±39.51 to 608.7±105.3 mg (*P*-value: 0.0027,^**^) (Figure 4C). This is likely to be due to the reduced tumour burden of PBOX-treated animals.

## Discussion

Our group has recently developed a novel set of compounds known as the PBOXs that actively induce apoptosis in numerous haematological cancers and solid tumours (Zisterer *et al*, 2000, 2001; Mc Gee *et al*, 2004; Mulligan *et al*, 2006). Representative compounds have recently been shown to induce apoptosis in *ex vivo* CLL patient samples including samples that are composed of cells with poor prognostic markers such as fludarabine-resistant cells with chromosomal deletions in 17p (McElligott *et al*, 2009). Furthermore, an *in vivo* study by our group has previously shown PBOX-6 to exhibit antitumour activity in a murine breast tumour model (Greene *et al*, 2005) highlighting their potential as novel anticancer agents. Recent data also suggest the PBOX compounds show activity in MDR cell lines by bypassing P-gp (Nathwani *et al*, 2009).

The tyrosine kinase inhibitor imatinib has revolutionised CML treatment since its regulatory approval in 2001 (Deininger and Druker, 2003). In spite of this success, disease progression into a fatal acute leukaemia still occurs. Disease progression is commonly associated with mutations within the kinase domain of Bcr-Abl that result in varying degrees of resistance to imatinib, these mutations have been identified as the major mechanism of acquired imatinib resistance. Therefore, the search for alternative drug treatments capable of overcoming such resistance is crucially important (O’Hare *et al*, 2007). For this reason, we analysed the effects of the PBOX compounds on CML cells harbouring such mutations.

The amino-acid mutations chosen for this study represented several functionally distinct kinase domain regions, including the P-loop (E255K), the site of a hydrogen bond with imatinib (T315I) and the activation loop (H396P) (Griswold *et al*, 2006). Transfected cells were first subjected to analysis by western blot and confirmed the presence of the Bcr-Abl protein. A viability assay showed that the mutant Bcr-Abl cells, displayed as expected, varying degrees of resistance to imatinib of between 2.9- and 11.1-fold for H396P and E255K cells, respectively, when compared to native cells. T315I cells showed complete resistance to concentrations of imatinib as high as 10 *μ*M. IC_50_ values and resistance fold difference could not therefore be determined for these cells. However, a previous report suggests an IC_50_ value of 18 *μ*M for Bcr-Abl-T315I cells treated with imatinib (Chen *et al*, 2006).

In comparison to the wide range of effects of imatinib on the viability of Bcr-Abl mutants, PBOX-6 and PBOX-15 reduced the viability to a similar extent in all four cell lines. The clones harbouring these mutations, particularly the prevalent mutations E255K and T315I, have been shown to exist before imatinib treatment (Roche-Lestienne *et al*, 2002, 2003; Shah *et al*, 2002; Hofmann *et al*, 2003; Kreuzer *et al*, 2003) and are associated with enhanced kinase activities and proliferation rates (Yamamoto *et al*, 2004; Griswold *et al*, 2006). Mutations such as E255K are also associated with significantly shorter survival rates than other mutations, regardless of their sensitivity to imatinib (Griswold *et al*, 2006), resulting in a more aggressive phenotype. This highlights the importance of the PBOX compounds in overcoming these aggressive mutations. The apoptotic effects of the PBOX compounds have also been shown to be irreversible, a recent study on the CML cell line, K562, has shown the PBOX compounds to continue to induce apoptosis 24 h after cells have been washed free of the compounds (Bright *et al*, 2010).

One of the most common Bcr-Abl mutations found in CML patients is the T315I mutation. Many imatinib-resistant mutants are sensitive to second-generation inhibitors such as dasatinib and nilotinib; however, the T315I mutation remains insensitive to both drugs (O’Hare *et al*, 2007; Quintás-Cardama and Cortes, 2008b). Thus, it was decided to concentrate our efforts on the T315I mutation for further analysis. Results showed PBOX-6 and PBOX-15 to induce similar levels of apoptosis in native and T315I mutant cells, suggesting that these agents are capable of overcoming imatinib resistance irrespective of Bcr-Abl genotype.

Although there are numerous reports of novel compounds effective at inducing apoptosis in T315I-expressing cells in the literature, many are at a pre-clinical stage such as ON012380 a non-competitive ATP inhibitor (Gumireddy *et al*, 2005), SGX393 a direct Bcr-Abl inhibitor (O’Hare *et al*, 2008) and the histone deacetylase inhibitor LAQ824 (Weisberg *et al*, 2004). Compounds that have displayed activity against the T315I mutants in clinical trials include the aurora kinase inhibitor MK-0457 (Giles *et al*, 2007) and homoharringtonine that inhibits protein synthesis (Quintás-Cardama and Cortes, 2008a). Other compounds currently undergoing clinical assessment but whose antitumour activity in patients has yet to be fully determined include the histone deacetylase inhibitors SAHA and LBH589, the farnesyltransferase inhibitor BMS-214662 and the aurora kinase inhibitors PHA-739358 and AT-9283 among others (Tanaka and Kimura, 2008; Quintás-Cardama and Cortes, 2008b). However, to date no compound with activity against the T315I mutation has made it through trials and onto the clinical market.

Although the effects of the PBOXs on Bcr-Abl mutations *in vitro* is clear, these results needed to be confirmed in *ex vivo* patient samples, critically important in predicting a clinical response to potential anticancer drugs. Of the newly diagnosed patients enlisted for PBOX evaluation, all *ex vivo* samples analysed (4 out of 4) displayed a significant increase in apoptosis after PBOX-6 and PBOX-15 treatment when compared with imatinib and control treatments. Half of these patients (2 out of 4) are currently on the front-line treatment for chronic phase CML, imatinib treatment, with the other half on nilotinib treatment as part of a clinical trial. These results suggest a potential role for the pro-apoptotic PBOX compounds in the treatment of newly diagnosed CML patients. Similar enhancements in apoptosis were also observed in two imatinib-resistant *ex vivo* samples both of whom tested positive for the T315I mutation. Although the low levels of the T315I mutation in patient 6 are unlikely to have contributed significantly to *in vitro* resistance, this patient also tested 100% positive for the F317l mutation, another highly imatinib-resistant mutation, suggesting the PBOX compounds also successfully induce apoptosis in cells harbouring this mutation. Although the number of primary samples obtained is limited and no firm conclusions can be drawn from the data of just two imatinib-resistant patients, these positive results do warrant a larger study assessing the effects of the PBOX compounds on further imatinib-insensitive primary samples to be undertaken.

Notably, the PBOX compounds do not display cytotoxicity in blood samples obtained from healthy volunteers (Mc Gee *et al*, 2004; Greene *et al*, 2007; McElligott *et al*, 2009). Furthermore, a low level of vasorelaxing activity and a lack of inhibition of nitrendipine binding, both measures of calcium antagonism suggest a general lack of cardiotoxicity of the PBOX compounds (DMZ, unpublished data). A lack of *in vivo* toxicity was also observed in unchallenged Balb/c mice administered for 8 days with PBOX-6 as determined by analysis of the haematology parameters of platelets, WBCs and red blood cells when compared to vehicle-treated mice (DMZ, unpublished data).

The PBOX compounds are not just specific for Bcr-Abl-positive cells but are also specific for a wide variety of cancerous cells. The molecular target of the PBOX compounds has recently been identified as tubulin and differences between the tubulin properties of cancer and non-cancer cells may therefore be responsible for the preference of the compounds for cancer cells. The compounds have been shown to bind to a novel, as yet uncharacterised binding site on tubulin (Mulligan *et al*, 2006) and work is currently underway to characterise this binding site using X-ray crystallography. This will give further insight into the exact mechanism of action and specificity of the compounds.

After the demonstration that the PBOX compounds induce apoptosis *in vitro* and in *ex vivo* patient samples, we next sought to determine the effects of the PBOX compounds in an *in vivo* CML xenograft model. The xenotransplant model has also been successfully used for the preliminary pre-clinical drug testing of SCH66336 and SXG393 (Peters *et al*, 2001; O’Hare *et al*, 2008) and although this model is not without limitations it does enable the effective testing of the compounds on the T315I mutation *in vivo.* Although PBOX-15 showed more potent activity in primary samples, PBOX-6 was chosen as a representative PBOX compound for *in vivo* testing as it has previously been shown to restrict tumour growth in an *in vivo* mouse mammary carcinoma (Greene *et al*, 2005). It remains to be seen whether PBOX-15 shows activity *in vivo*. Female Balb/c nude mice were injected s.c. with Baf/3 T315I CML cells and randomly divided into two treatment groups. Results showed that PBOX-6 significantly impeded tumour growth while also preventing gross spleen enlargement when compared to mice receiving vehicle injections. Mice did not experience weight loss or exhibit any signs of distress during PBOX treatment suggesting PBOX-6 to be a viable anticancer agent *in vivo*. Although the control group also appeared to gain some weight throughout the experiment, this may have been due to the increase in spleen weight rather than natural growth.

Although tumour growth was significantly reduced, the tumour mass did still increase over the duration of the experiment; however, the results from this and previous research suggest the true potential of the PBOX compounds *in vivo* could lie in their combination with other anticancer compounds, recent data have shown the ability of the PBOXs to synergise with other compounds including imatinib and the CDK1 inhibitor flavopiridol. (Greene *et al*, 2007; Bright *et al*, 2009, 2010). Future studies looking at the activity of the PBOX compounds in combination with these agents *in vivo* are planned, as are advanced models that more readily replicate the disease *in vivo*, such as the retroviral CML mouse model. Ongoing work with a collaborating laboratory, looking at the design and synthesis of ‘second-generation’ more potent PBOX compounds could potentially further increase the activity of the compounds.

In conclusion, the PBOX compounds significantly reduced the cell viability of CML cells lines harbouring the E225K, H396P and T315I Bcr-Abl mutations. Both PBOX-15 and PBOX-6 significantly increased the number of cells undergoing apoptosis in native and T315I cells *in vitro* and in *ex vivo* patient samples whereas PBOX-6 also significantly reduced tumour burden in a mouse xenograft model. These results underline the potential of the PBOX compounds in the clinical treatment of CML patients including those who develop the highly resistant T315I mutant.

## Figures and Tables

**Table 1:** Characteristics of CML patients in *ex vivo* study

**No.**	**Sex**	**Age**	**Disease status**	**CML treatment**	**Current status**
1	M	46	Newly diagnosed	Imatinib	Currently on imatinib, CCR within first year treatment
2	F	31	Newly diagnosed	Imatinib	Currently on imatinib, CCR within first year treatment
3	M	26	Newly diagnosed	Nilotinib (as part of clinical trial)	Currently on nilotinib, MCR in first 3 months treatment
4	F	42	Newly diagnosed	Nilotinib (as part of clinical trial)	Currently on nilotinib, MCR in first 3 months treatment
5	F	73	Blast crisis	Failed imatinib+dasatinib treatment	Deceased, 100% positive for T315I mutation at time of death
6	F	62	Blast crisis	Failed imatinib+dasatinib treatment	Currently on nilotinib, 100% positive for F317I mutation and minor T315I clone at time of sample collection

Abbreviations: CCR=a complete cytogenetic response means that no blood or bone marrow cells contain the Ph chromosome; CML=chronic myeloid leukaemia; M=male; F=female; MCR=a major (or partial) cytogenetic response the presence of less than 35% Bcr-Abl-positive cells.

Relevant medical and personal data were obtained from newly diagnosed and imatinib-resistant CML patients enrolled in an *ex vivo* PBOX study carried out in St. James's Hospital, Dublin 8 in collaboration with Dr. Eibhlin Conneally and Professor Mark Lawler. Information obtained included the sex of the patient, age at diagnosis and a short description of their medical and CML-specific treatment.
